# Biological Materials for Tissue-Engineered Vascular Grafts: Overview of Recent Advancements

**DOI:** 10.3390/biom13091389

**Published:** 2023-09-14

**Authors:** Dalila Di Francesco, Alexa Pigliafreddo, Simona Casarella, Luca Di Nunno, Diego Mantovani, Francesca Boccafoschi

**Affiliations:** 1 Department of Health Sciences, University of Piemonte Orientale “A. Avogadro”, 28100 Novara, Italy; dalila.di-francesco.1@ulaval.ca (D.D.F.); simona.casarella@uniupo.it (S.C.); 20027949@studenti.uniupo.it (L.D.N.); 2 Laboratory for Biomaterials and Bioengineering, CRC-I, Department of Min-Met-Materials Engineering, University Hospital Research Center, Regenerative Medicine, Laval University, Quebec City, QC G1V 0A6, Canada; diego.mantovani@gmn.ulaval.ca

**Keywords:** vascular tissue engineering, natural biomaterials, tissue-engineered vascular grafts

## Abstract

The clinical demand for tissue-engineered vascular grafts is still rising, and there are many challenges that need to be overcome, in particular, to obtain functional small-diameter grafts. The many advances made in cell culture, biomaterials, manufacturing techniques, and tissue engineering methods have led to various promising solutions for vascular graft production, with available options able to recapitulate both biological and mechanical properties of native blood vessels. Due to the rising interest in materials with bioactive potentials, materials from natural sources have also recently gained more attention for vascular tissue engineering, and new strategies have been developed to solve the disadvantages related to their use. In this review, the progress made in tissue-engineered vascular graft production is discussed. We highlight, in particular, the use of natural materials as scaffolds for vascular tissue engineering.

## 1. Introduction

Cardiovascular diseases (CVDs) are a group of pathologies that affect the cardiac and vascular systems; they include coronary heart disease, cerebrovascular pathologies, rheumatic heart disease, and other conditions. CVDs are the main cause of death in the world, with 17.9 million deaths per year, according to the World Health Organization (WHO). Almost 85% of CVD-related deaths are caused by heart attack and strokes, and a third of these deaths involve individuals under 70 years of age [1].

The main cause of these pathologies is atherosclerosis, which is a progressive condition characterized by the formation of atherosclerotic plaques that develop in the intima layer of blood vessels and lead to the partial or total obstruction of vessels [2]. The most common treatment is pharmaceutical therapy, coupled with a healthy lifestyle and balanced diet; however, in the case of occlusive CVDs, the ultimate treatment options are surgical, represented by vascular stents, substitution surgery, or vascular bypass. The latter solutions aim at replacing the damaged vessel or at redirecting blood flow around it through the use of vascular grafts [3]. For these purposes, the main source of vascular grafts are autologous blood vessels, such as the saphenous vein or the internal mammary artery, which, for example, represents the gold standard for coronary artery bypass surgery. Autologous blood vessels are naturally biocompatible, non-thrombogenic, and have the necessary mechanical properties to suit vascular application [4]. However, this treatment option is still hampered by different problems, the main one being implant failure, which occurs, for example, in around 50% of patients 10 years after a saphenous vein graft implant [5]. Media layer hyperplasia or vein graft disease can also arise after implantation, which lead to the occlusion of the lumen and implanted graft [6,7,8]. Moreover, the use of autologous blood vessels is not always an option, due to either the multiple surgical procedures required, the patients’ age and health conditions, or the mismatch of blood vessel dimensions [9].

As an alternative, commercial vascular grafts can be used. Most of these are usually made from synthetic materials such as polyethylene terephthalate (PET) and polytetrafluoroethylene (e-PTFE); however, some biological commercial products can also be found. LeMaitre Vascular produces natural commercial grafts such as Artegraft^®^, a xenograft from bovine collagen, ProCol^®^, a bioprothesis from the bovine mesenteric vein, or Omniflow II^®^, which is a biosynthetic graft. Bioprotec S.A.S.U. produces human-derived vascular grafts from the saphenous vein which range from small- to medium-caliber applications. Even though there are commercial alternatives to autologous bypass, they still present some limitations: their mechanical properties are not always appropriate and they may cause aneurysms and/or thrombosis, allergic reactions, and, generally, have a high implant failure rate. Another challenge is represented by patency: synthetic grafts show good patency rates in the case of large-diameter grafts, e.g., 85% patency at 5 years after implantation for PET, but patency rates decrease in the case of small-diameter vascular grafts. On the other hand, biological alternatives still require advancements to achieve the results achieved with synthetic grafts but show promising potential; for example, Artegraft^®^ solution was able to achieve 73% patency 18 months after implantation [10,11,12,13,14,15,16,17].

The success of the existing approaches is still very much constrained by the aforementioned problems; thus, valid alternatives to obtain functional vascular conduits are needed. One approach that represents a potential solution is vascular tissue engineering (VTE), a branch of regenerative medicine that aims at producing innovative solutions to substitute damaged blood vessels. To do so, VTE relies on the production of tissue-engineered vascular grafts (TEVGs), which are made in vitro by combining cells, biomaterials, fabrication techniques, and tissue maturation methods [18,19].

Since the first ever TEVG reported in 1986 [20], a better understanding of these components and the progress made in the technologies used has permitted huge advancements in the making of TEVGs. Appropriate mechanical and biological properties for TEVGs’ success are difficult to achieve, and grafts’ failure greatly depends on the choice of cells, biomaterials, the technique used to manufacture the graft, and how these parts interact with each other. In particular, innovations in biomaterial production and characterization have allowed high-quality bioactive materials for VTE to be produced; these have improved the success of TEVGs [21,22]. This review focuses on evaluating the latest advances made in natural-based biomaterials and their fabrication techniques for VTE.

## 2. Vascular Tissue Engineering Requirements and Fundamentals

**Figure 1:**
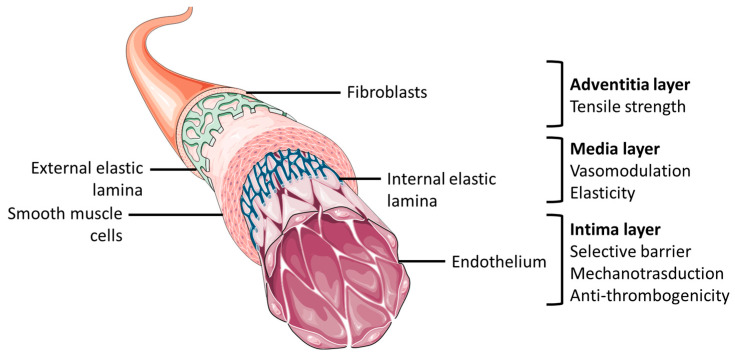
General anatomical structure of blood vessel and layers’ functional characteristics. The figure was partly generated using Servier Medical Art, provided by Servier, licensed under a Creative Commons Attribution 3.0 unported license.

TEVGs are designed as alternatives to substitute for damaged blood vessels, and, thus, they should have specific requirements that allow them to closely mimic physiological conditions [23]. Native blood vessels are made of a concentric three-layered structure referred to as the intima, media, and adventitia layers. The intima layer is constituted of an endothelial cell (EC) monolayer adhered on a basal membrane. The adventitia and media are three-dimensional layers characterized by cells, fibroblasts (FB), and smooth muscle cells (SMC), respectively, embedded in extracellular matrix (ECM) [24]. This structure allows for the main biological and mechanical functions of blood vessels (Figure 1).

Thus, an ideal graft should have adequate biological and mechanical characteristics [14,25]. On the biological side it should be noncytotoxic, nonimmunogenic, non-thrombogenic and hemocompatible. Moreover, it should allow cell repopulation and remodeling [9]. Mechanically, it is of utmost importance that the vessel is able to bear blood pressure, without any damage or permanent deformation. Furthermore, compliance must be considered: the graft should be able to adjust to blood pressure changes without creating stress or turbulent flows. It should be viscoelastic, allowing the right balance between elasticity and mechanical strength and maintaining these properties over time. Graft patency is another factor to consider, as maintaining patency, especially in small-caliber vascular grafts, is still a challenge in VTE [26]. Finally, it should also be able to sustain the surgical grafting process, which involves stitching [16,27,28].

**Figure 2:**
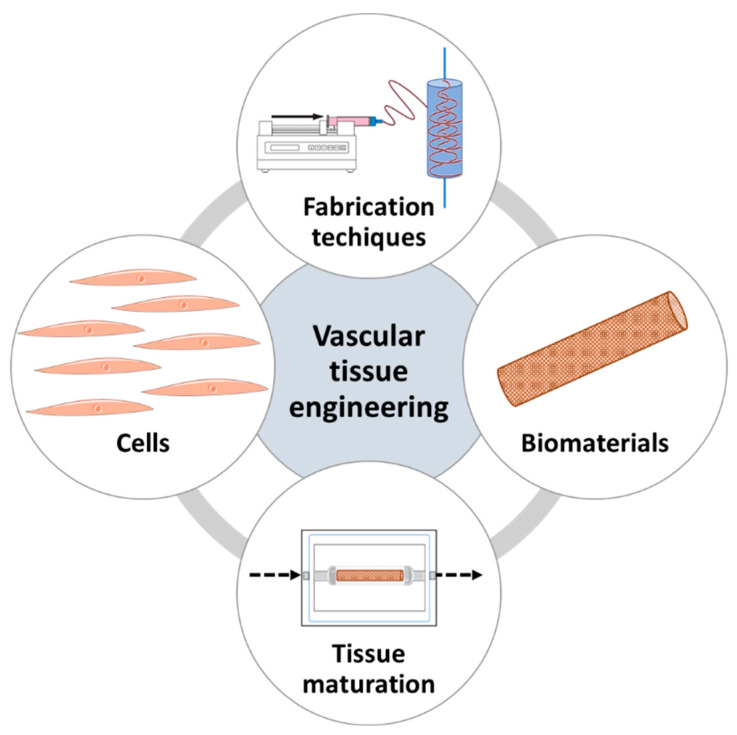
The fundamentals of vascular tissue engineering.

In order to achieve these complex properties and fully recapitulate the physiological environment, appropriate cell sources, optimal biomaterial, and fabrication and maturation techniques are important elements to consider; as a result, these constitute the fundamentals of VTE (Figure 2). Understanding these elements and the rationale behind the choice is essential; thus, a synopsis of each of these components will follow [19,27].

### 2.1. Cell Sources in Vascular Tissue Engineering

Cells are of utmost importance to reproduce vascular tissue and ensure structural stability. The appropriate cell source choice should also facilitate in vivo integration of the TEVG. The cellular environment should represent the trilayered structure of blood vessels; therefore, ECs, SMCs, and FBs are used. Different cell lines and sources have been evaluated over time, both from primary commercial human lines—mainly used for basic research purposes—or stem cells isolated from different tissues. However, problems can arise from the tissue used for stem cell source isolation and in the amount of cells isolated, which is often unsatisfactory [19,29].

Among the first cell types studied were human primary cells. These cells have the advantage of being isolated in a tissue-specific manner, and they allow rejection-related problems to be reduced. However, these cells are difficult to isolate, and it is difficult to obtain adequate yields and maintain the differentiated phenotype [30]. Due to their unlimited self-renewal ability, stem cells represent a good option to overcome this problem. Autologous adult stem cells can be used, but their adequacy depends on the patient’s age. Although their application is limited by ethical problems and potential tumorigenic threats, embryonic stem cells are also being used [31]. Other options include induced pluripotent stem cells (iPSCs) [32], which allow autologous transplant, and genetic engineering approaches to reprogram somatic cell lines [30].

### 2.2. Biomaterials in Vascular Tissue Engineering

The choice of biomaterial is one of the key elements in achieving a successful TEVG. In fact, biomaterials’ properties play a major role in dictating the mechanical properties of the graft; furthermore, the efficacy of the biomaterials’ interaction with the cells drives the formation of effective vascular like-tissue. Physiologically, vascular cells are embedded in the extracellular matrix, which is responsible both for creating the appropriate mechanical environment and providing the necessary biological cues. Thus, the aim of the biomaterial choice is to mimic this natural environment as closely as possible. In particular, for VTE, biocompatibility, hemocompatibility, nonimmunogenicity, bioactivity, architecture, degradability (when required), and physical characteristics must be considered [33,34].

Biomaterials for VTE can be processed and used in different forms such as sheets, decellularized scaffolds, hydrogels, bioinks, or electrospun scaffolds [23,27]. Both synthetic, natural, and a combination of both biomaterial types can be used. On the one hand, synthetic materials have the advantage of being easily tailorable; thus, they allow controlled mechanical properties to be obtained in a reproducible manner. However, they often lack biological cues. On the other hand, natural materials commonly show excellent biocompatibility and bioactivity, but they are difficult to tailor, display weak mechanical properties, and often demonstrate batch-to-batch variation. For these reasons, combining both sources often proves advantageous [21,35,36].

Synthetic materials such as polytetrafluoroethylene (ePTFE), polycaprolactone (PCL), polylactic acid (PLA), and polyglycolic acid (PGA) are commonly used for VTE. These materials offer great mechanical properties; however, they have low permeability, do not efficiently promote material–cell interactions, and often induce an inflammatory cascade [22,37]. Research is currently moving towards the use of bioactive materials, meaning biomaterials that are not simply biocompatible but that also interact with the environment in which they are implanted, producing a beneficial response. Thus, synthetic materials can be conveniently tailored, or, simply, natural materials can be used [38]. For VTE, frequently used materials include collagen, elastin, fibrin, gelatin, hyaluronic acid, silk, chitosan, and decellularized extracellular matrix [39].

### 2.3. Fabrication Techniques

The manufacturing technique used to make TEVGs is an important factor to consider in VTE design. Depending on the technique selected, this may permit one to achieve a certain architecture of the scaffold, which can be tailored to obtain specific characteristics such as tubular geometry, precise porosity and pore size, and/or fiber orientation [40]. Moreover, the choice is closely dependent on the biomaterial selected, and vice versa [39]. The manufacturing strategy can range from relatively simple, like cell sheet engineering, to much more complex, like electrospinning [41]. In any case, the aim is to be able to find a method that allows one to attain structural properties that closely resemble the 3D physiological environment, allowing for the distribution, organization, and maturation of cells in the scaffold in a reproducible and scalable way. The techniques can be divided into cell-free scaffolds, onto which cells are subsequently seeded, or cell-laden scaffolds, meaning manufactured in the presence of cells [42]. Cell sheet engineering, decellularization of xenogeneic tissues, molding, electrospinning, and 3D printing are among the most used techniques; thus, a summary of what they are and their application in VTE will follow.

#### 2.3.1. Cell Sheet Engineering

The traditional technique of cell sheet engineering is part of a wider group of strategies called tissue engineering by self-assembly (TESA). This method consists in rolling together different cell sheets around mandrels or molds, one by one, until the desired construct is obtained [43]. There are several approaches, beginning with the standard technique: cells are cultured forming bidimensional sheets, which are then wrapped around a mandrel. In other cases, the sheet fabrication is supported by cells cultured onto a biomaterial scaffold. Finally, there is the stacking method, in which different cells sheets are assembled layer by layer, allowing one to understand the interactions between the different cell types [44,45,46].

In 1998, Heureux et al. pioneered the use of the stacking technique to produce a TEVG. They were able to culture SMCs and fibroblasts in order to compose layers formed by cells entrapped in ECM. After a month of culturing, these cellular layers could be retrieved and wrapped around a tubular mandrel to obtain a tubular multilayered cellular construct. Including tissue maturation, this technique took almost 3 months to achieve the final vascular graft [47]. With time, TESA techniques have been optimized, as described by Torres et al. in 2021. In fact, they were able to obtain cell-assembled ECM sheets by fibroblasts derived from different species, demonstrating how the species led to different properties of the products, such as different fibroblast behavior in proliferation, maturation, and structural arrangement, allowing for the selection of the best fit for future TEVG production with the cell-assembled sheets [48]. Magnan et al. also brought advances to the field of cell sheet engineering, as they obtained yarns from human fibroblasts’ cell-assembled extracellular matrix sheets, which were then used to fabricate TEVGs with mechanical properties superior to physiological vessels and other natural TEVGs [49]. Additionally, the method of cell-sheet engineering has led to the production of different grafts which have reached clinical trials [50,51].

#### 2.3.2. Decellularized Vessels

The use of decellularized extracellular matrix in VTE is a common strategy as it allows for the exploitation of the native properties of the vessel while maintaining the mechanical characteristics and without inducing adverse immune reactions [52]. In this context, blood vessels are usually harvested from different animal sources then decellularized with enzymatic, chemical, and/or physical techniques with the aim of eliminating all cellular components while maintaining the physiological structure and properties [53,54,55]. The TEVGs can then be used as acellular scaffolds or implanted after recellularization with autologous cells [56,57,58].

This technique, indeed, ranges back several years, with many in vitro and in vivo studies. For instance, Badylak et al. produced TEVGs from decellularized small intestine submucosa which were applied as large-diameter vascular infrarenal aorta grafts in dog models, with survival up to 52 weeks post-implantation, as far back as 1989 [59]. In recent years, numerous studies have been performed to optimize the techniques, including functionalizing the decellularized grafts for better recellularization and maintenance of biomechanical properties [60,61,62,63].

#### 2.3.3. Molding

The molding method consists in pouring a biomaterial solution, with or without cells, into a mold designed to replicate the desired structure. Once the solution solidifies and/or gelifies, the mold is removed and the desired tubular construct is obtained [64,65]. This strategy is relatively easy, cheaper, and timesaving compared to others; in addition, it has commonly been employed in VTE, producing advancements in the technique.

In 2007, Boccafoschi and colleagues were able to obtain a collagen-cell tubular TEVG with encouraging but not adequate mechanical properties and excellent biological performance using a simple, custom-made rotating device [66]. More recently, Helms et al. produced a trilayered bioartificial blood vessel using a step-by-step casting technique. They used fibrin hydrogels as a layer base, cellularized with SMCs for the middle layer, ECs, and adipose-derived stem cells as the outer layer. Finally, the internal layer was generated by seeding ECs in the luminal side. They obtained TEVGs with a physiological-like, trilayered architecture with this method [67].

#### 2.3.4. Electrospinning

Electrospinning is a relatively innovative and advanced technology that allows for the fabrication of diverse structures through the principle of jet-extruding the electrified solution, which is then elongated into fibers stretched on the collector. Although a more expensive and complex technique, electrospinning allows for the formation of nanofibrous scaffolds with very controlled morphology, as the size, diameter, hierarchical arrangement, and homogeneity of the fibers are parameters that can be controlled [68,69]. In particular, for VTE, this strategy allows one to obtain tubular scaffolds with controlled mechanical properties and has been heavily employed in recent years for the production of TEVGs [70].

In 2019, Niu et al. were able to obtain vascular grafts possessing the vascular vessel property of nonlinear elasticity, the stability of which was maintained under stresses, thanks to mechanical stimulation performed on finely controlled electrospun fiber alignment. Furthermore, the fiber alignment also allowed for cell guidance orientation [71]. In 2021, Do et al. produced bilayered electrospun vascular grafts which presented a physiological-like structure. Thanks to the nanoscale fibers of the inner layer, good endothelialization on the graft was achieved. Moreover, the overall structure allowed the TEVG to withstand vascular forces due to its ability to maintain appropriate mechanical properties [72].

#### 2.3.5. 3D Printing

A quite complex fabrication strategy is represented by 3D printing. This technology allows one to extrude bioinks in a layer-by-layer approach, in the presence of cells and bioinks, or acellular, into a defined structure previously modeled by computer graphics. Aside from its main advantage—the possibility to print live cells embedded in the scaffold—this strategy also allows for the creation of finely shaped and designed structures with complex geometries which closely mimic the physiological structure of ECM. However, the main challenge of 3D bioprinting is the choice of material, as the material should have particular rheological properties in order to be an extrudable material [73,74]. For these reasons, it has become a strategy of interest for VTE and many advancements have been made in the past years in the research of suitable bioinks and the production of 3D-printed TEVGs [75].

In 2019, Freeman et al. were able to produce a bioink from fibrinogen, an optimal biomaterial for VTE but usually difficult to print. Their results showed that the bioink, coupled with a new rotary printing method, created cellularized TEVGs with increased mechanical properties and collagen-producing cells [76]. Moreover, Zhou et al. made bilayered cell-laden TEVGs using coaxial bioprinting. They demonstrated that their novel bioink was able to create a porous 3D-printed cylinder that favored SMC proliferation while maturing. Moreover, the TEVGs showed optimal mechanical properties under physiological flow simulation [77].

### 2.4. Tissue Maturation

During tissue maturation, cells are cultured in their 3D environment, and, with adequate stimuli, they should establish cell–cell and cell–biomaterial interactions, proliferate, and synthetize new extracellular matrix; for this reason, tissue maturation represents a critical process in VTE [78]. Tissue maturation can occur in static (passive, with traditional cell incubators) or dynamic conditions; the latter is usually performed by the use of bioreactor systems designed to provide different mechanical stimuli, like shear stresses, pressure control, and flow rates, which simulate physiological hemodynamic stimuli. For a review on the subject, see the work of Mitchell et al. [79]. Dynamic vascular graft maturation has proven, time and time again, to be able to produce more mechanically and biologically appropriate properties [80,81]. Moreover, bioreactors can also be used to perfuse the grafts with cells, for example, with the purpose of endothelialization [82,83].

These four components of VTE are key in obtaining TEVGs with appropriate properties, and over the years, many improvements have been made in their employment to obtain vascular grafts that are more able to mimic the natural vessel environment. In Table 1, recent examples of how these components have been employed for VTE are shown along with the major findings of these studies.

## 3. Natural Biomaterials for Vascular Tissue Engineering

The success of a TEVG greatly depends on the choice of the biomaterial used. As previously mentioned, the biomaterial should have some key features in order to appropriately fulfil its use, and, as of late, bioactivity has been regarded as one of the most important biomaterial properties for tissue engineering applications [98]. For these reasons, naturally derived biomaterials have become of great interest, as they provide the necessary biological cues to interact and guide cell functions, while synthetic materials, although easy to tailor, remain almost inert if not appropriately functionalized. Natural materials are usually classified according to whether they are protein-based, polysaccharide-based, or decellularized extracellular matrix. These biomaterials display biocompatibility, bioactivity, and biodegradation, and are mostly nonimmunogenic; however, they are difficult to fine-tune, show batch-to-batch variability, and commonly display weak mechanical properties [99,100,101].

In particular, for VTE applications, the appropriate interaction between the material and the blood interface is a necessary feature for a TEVG’s success, together with the endothelialization process and the control of SMCs’ proliferation and functionality. Thus, the domains found in naturally derived materials are suitable for this, as they allow for cell recognition and adhesion [3]. In recent years, the use of natural materials as vascular graft scaffolds has been greatly explored. Commonly, collagen, gelatin, fibrin, elastin, silk, chitosan, and decellularized extracellular matrix are used, and they will be briefly reviewed hereafter.

### 3.1. Collagen

Collagen is the most abundant protein in the body, representing a third of total proteins and the main component of ECM. Among the 28 types of collagen identified, collagen type I is most used as a biomaterial [102,103]. The use of collagen in tissue engineering is common due to its many advantages such as biocompatibility, cell adhesion sites, biodegradability, nonimmunogenicity, availability, and hydrophilicity. Collagen is the most commonly used natural material for VTE, as it is the main ECM component of the vascular wall, responsible for load carrying and pressure resistance [104]. Its use for vascular graft fabrication dates back to 1986 [20], and, with time, more complex TEVGs were fabricated with collagen as the core biomaterial. Aside from being used as the core material for TEVG scaffolding, it has also been historically used for VTE as a biomimetic coating and drug delivery system [104,105,106].

In 2022, Camasão et al. produced cellularized collagen trilayered vascular grafts. In their study, the grafts were composed by molding two layers of cellularized collagen with fibroblasts and SMCs, respectively, then creating an endothelial layer using a homemade rotating system. Furthermore, the construct was cultured under physiological mechanical stimuli. This method proved to improve both cell alignment, remodeling, and EMC production, along with the scaffold’s viscoelastic properties, when compared to grafts matured in static conditions, thus producing a reliable in vitro model of the vascular wall [107]. In another study, Bosch-Rué et al. prepared bilayered cellularized collagen TEVGs with a coaxial extrusion method. For this purpose, highly concentrated collagen was used, containing SMCs for the outer layer and ECs for the inner layer; the extruded construct showed the ability to withstand physiological mechanical properties such as pressure and flow rate [108]. Justin et al. created a new, simple, and cheaper method to mold densified collagen tubular constructs for VTE, with controllable geometry and surface patterns. This strategy allowed customizable collagen constructs with superior mechanical properties and stability, due to densification, to be obtained. Moreover, they showed that the method and densified collagen was adequate for cell culture, allowing high concentration cellularization with different cell lines [109].

These works show how the progress in manufacturing techniques and tissue maturation methods has allowed collagen constructs with better mechanical properties to be achieved. These achievements are considerable, as collagen, although highly biocompatible and bioactive, demonstrates weak mechanical properties in terms of its ability to sustain blood flow and pressure. Another common method to achieve collagen grafts with superior mechanical properties is to hybridize it with synthetic polymers.

In 2022, Ma et al. studied the advantages of adding collagen to a PCL/heparin construct. In particular, their electrospun collagen/PCL/heparin composite TEVGs showed not only native-like mechanical properties but, also, cell compatibility thanks to collagen’s biocompatibility and ability to avoid structural deformation under stretching. Furthermore, the presence of collagen allowed the controlled release of heparin, favoring tissue regeneration [110]. A study by Jia et al. evaluated the presence of electrospun collagen and hyaluronic acid in enzyme-laden PCL TEVGs. Their work demonstrated that the use of collagen enhanced endothelialization of the graft, and that the synergy with enzyme enrichment also allowed antithrombotic properties to be achieved, both in vitro and in vivo, maintaining patency up to a month after being implanted in rat models [111].

Even though collagen still presents limitations—such as weak mechanical properties, compared to synthetic polymers, and thrombogenicity—due to the excellent biological, potential collagen is still one of the most interesting natural materials for TEVG production. Moreover, thanks to the advancements achieved in improving its mechanical properties and tailorability, the use of collagen in VTE is still constantly increasing.

### 3.2. Gelatin

Gelatin is a material derived from the denaturation of collagen’s triple helix. Thanks to its biocompatibility, biodegradability, low cytotoxicity, immunogenicity, and, finally, low cost, gelatin has been widely used as a biomaterial for tissue engineering. However, a disadvantage of gelatin is related to the need for chain reticulation in order to maintain its stability. For this reason, it is mainly used in the functionalized form: gelatin methacryloyl (GelMA). To stabilize this form, it is often reticulated with other materials, allowing for an increase of degradation time and improvement of its water resistance. GelMA has been used in combination with other materials to improve their biological performance for TEVG production [24,112].

Thus, GelMA requires advanced methods in manufacturing to be used alone. In 2021, Peng et al. prepared an enhanced GelMA construct for VTE purposes. They demonstrated how their 3D-printed GelMA could be made stronger and tougher using a novel dual-cross-linking method after 3D printing. Moreover, the biological performances of GelMA were preserved, as ECs perfused in the inner layer of the tubular construct showed adequate cell adhesion and viability [113]. Fazal et al., in 2023, studied additive lathe printing as a method to make long, helical-structure, tubular grafts from GelMA hydrogels. Their study shows how the method allows more control of the geometry of the constructs, which showed physiological anisotropic characteristics and resistance to burst pressure [114].

Another way to achieve gelatin’s stability is to combine it with other materials, either natural or synthetic. For example, Joy et al. evaluated the cross-linking of gelatin using oxidized carboxymethyl cellulose to make tunable electrospun TEVGs. With optimal electrospinning parameters, they were able to obtain constructs that showed great biocompatibility and low immunogenicity after in vivo implantation in rats, although they still degraded rapidly; thus, use in combination with other synthetic materials was proposed [115]. Huang et al., in a preliminary study, prepared a bilayered scaffold by electrospinning PCL, polyethylene glycol (PEG), and gelatin. After achieving the optimal material blend for ultrastructure properties, cocultures of SMCs and ECs seeded on the membranes were set up, and the results showed good endothelialization on the surface, cell adhesion, and migration, with the 3D colonization of SMCs on the scaffold [116]. In 2020, the same research group evaluated, instead, a blend of PCL, PLGA, and gelatin for electrospinning. The new blend’s fiber orientation, coupled with the properties of gelatin, improved the mechanical properties of the scaffold. The biocompatibility traits were maintained, and guided cell orientation on the scaffold was also achieved [117].

Although it is a cheap biocompatible option, gelatin still has the disadvantage of requiring modifications and cross-linking in order for one to obtain optimal stability. However, its use in combination with other materials for VTE is promising to enhance biological properties and endothelialization.

### 3.3. Fibrin

Fibrin is the active and insoluble form of fibrinogen, a protein involved in the coagulation cascade and wound healing. Because of its adhesive properties, it is widely used as a sealant in biomedical applications [118]. Moreover, it is an interesting material for scaffold design, both in gel and fiber form. Fibrin can be isolated from a patient’s plasma, representing an interesting option in terms of personalized tailored biomaterials, limiting immunological reaction risks [45]. Although fibrin is another natural material that does not possess appropriate mechanical properties for TEVGs’ production, it is still used for VTE because fibrin fibers can closely replicate the structure of ECM and guide cell functions and remodeling [119]. Therefore, it is frequently found used in combination with other materials.

In 2020, Yang et al. prepared electrospun TEVGs made of polyurethane (PU) and fibrin with small-diameter fibers. PU was used in order to increase the stability and mechanical properties of the fibrin scaffold; once the proper combination of materials was achieved, in terms of mechanical strength, biocompatibility, and hemocompatibility, in vivo experimentation using rat models was conducted. The results showed good patency and mechanical properties up to 3 months after implantation, while the presence of fibrin promoted the biological functions, avoiding thrombosis and graft occlusion and enhancing cell guidance and ECM deposition [120]. The same research group also evaluated the use of PCL to enhance the mechanical properties of fibrin. The electrospun PCL/fibrin scaffolds showed different ultrastructures, leading to small-diameter grafts with good water absorption and increased tensile strength. Moreover, the TEVGs demonstrated good hemocompatibility and biocompatibility, tissue remodeling, and degradability in vivo using rat models [121]. In fact, they evaluated the performance of the TEVG in vivo up to 9 months in rats. Their results proved the ability of the graft to induce endothelialization and cell repopulation of the scaffold, with few calcification areas. The TEVG also led to a reduction in the inflammatory response, inducing a regenerative process instead, guiding the regeneration of neoarteries [122].

Thrombogenicity and off-the-shelf availability are problems related to vascular grafts; however, Elliott et al. were able to overcome this problem by embedding anticoagulant heparin in fibrin scaffolds, which showed stability after up to 12 months in storage. The TEVGs maintained mechanical stability, ability to deliver the anticoagulant, and biocompatibility after implantation; however, graft occlusion at 5 weeks after implantation was still an issue [123].

Another example of fibrin’s use in TEVG production is from Syedain et al. In 2014, the research group published a work demonstrating the success of decellularized fibrin-based small-caliber TEVGs, produced by culturing and dynamically maturing ovine fibroblasts on fibrin scaffolds which were then decellularized. The grafts were then implanted in an ovine model and evaluated at 2 and 6 months after implantation. Their work demonstrated that the all-natural TEVGs remained patent up to 6 months, with evidence of endothelialization, cellularization, and cell-based remodeling of the grafts [124]. In 2017, the same TEVGs were produced by the research group, using human fibroblasts instead, implanted in baboon models, and evaluated at 1, 2, 3, and 6 months after implantation. In this case also, the TEVGs demonstrated recellularization after explantation, a promising patency rate of 60% at 6 months, and no adverse immune responses, suggesting the possibility of future clinical trials [125].

Fibrin undoubtedly demonstrates many advantages for TEVGs’ production, especially because of the possibility of obtaining it from patients’ blood. However, the studies performed still use it in combination with other materials in order to achieve the necessary mechanical properties.

### 3.4. Elastin

Elastin is a protein of the connective tissue responsible for tissue elasticity. Tropoelastin, the soluble precursor of elastin, links together in an autonomous process to form an insoluble network of elastic fibers. These fibers provide recoil to tissues that undergo stretching forces. In particular, in the vascular tissue, elastin plays both a mechanical and biosensing role, allowing for elastic expansion and contraction. Moreover, elastin plays a role in the inhibition of SMCs’ hyperproliferation and has antithrombotic properties [126]. As a biomaterial, elastin has been widely used, especially for VTE, since it is a major component of blood vessels’ ECM, playing a key role in its mechanical properties. However, it is difficult to use because of its highly reticulated nature and insolubility; thus, it is mainly used in its soluble forms, like tropoelastin, alpha-elastin, elastin-like polypeptides (ELPs), and elastin-like recombinamers (ELRs). Also, it is used in combination with other materials in order to provide them with an elastic component [127,128].

In a 2023 study, Natsume et al. synthesized a new type of ELP block copolymer with the ability to self-assemble into fibers. Moreover, to enhance bioactivity, endothelial cell adhesion peptide sequences were added. Their material proved to improve functional endothelialization while maintaining the contractile phenotype of SMCs without their overactivation; in addition, it limited platelet adhesion. Thus, their new material proved to meet all the biological requirements for TEVGs’ production [129].

Collagen and elastin are also often used in combination for VTE, as they are both major components of blood vessels. In particular, elastin is used to provide elasticity to the scaffold. These types of scaffolds present higher porosity and structural features that promote their use for small-caliber TEVGs, while stimulating endothelialization and preventing SMCs’ hyperplasia [24].

Ryan et al. proposed a new multistep technique for multilayer collagen/elastin TEVG fabrication. The proposed method combined solvent evaporation, rolling the sheet over a mandrel for tubular geometry, cross-linking, and, finally, lyophilization of the scaffold. The results showed a highly tailorable graft in terms of ultrastructure, closely mimicking physiological features, with optimizations performed on each layer of the graft. The grafts were then tested for 21 days in vitro with SMCs, showing good cell integration with remodeling into a dense vascular wall, low immunogenicity, and control over elastin’s density and location in the graft [130]. In another work, Camasão et al. attempted to enhance the elastic properties of cellularized collagen vascular scaffolds by adding bioactive forms of ELRs. The presence of ELRs induced cellular remodeling, compacting the construct and improving mechanical properties such as elastic modulus and tensile strength at break. Moreover, cellular proliferation and ECM deposition were also increased in the presence of ELRs, confirming the important role of elastin in the production of native-like grafts [131].

Aside from collagen and elastin composites, elastin has been widely reported to increase the elastic properties of TEVGs in combination with other materials. For example, in 2021, Tanaka et al. developed vascular grafts starting from insoluble elastin mixed with silk fibroin using a knitting machine. The addition of elastin to the scaffolds showed decreased platelet adhesion on the scaffold and increased EC adhesion instead. Moreover, the TEVGs showed the prevention of blood leakage, maintaining mechanical properties and patency after in vivo implantation in rats and promoting EC cell migration on the graft [132]. Instead, Wang et al. used a tropoelastin and polyglycerol sebacate (PGS) blend for electrospinning nonporous TEVGs. The stable scaffolds showed great biocompatibility both in vitro and in vivo. In particular, in vivo studies in mouse models showed that the graft decreased thrombosis risk, and new intima and adventitia layers were produced 2 months after implantation, with ECM deposition, cell integration in the layers, and appropriate structure. By 8 months, the scaffold had degraded, leaving a new artery formed [133].

The main disadvantage of elastin as a scaffold is the insolubility; however, with recent advancements in integrating and manufacturing derivatives, it was widely used for TEVGs’ production, demonstrating the ability to improve mechanical and biological properties and decreasing platelet adhesion.

### 3.5. Silk

Silk is a versatile biopolymer mainly produced by insects, such as larvae, and by spiders. This natural material is extremely resistant to traction, aside from being very biocompatible, and has, therefore, been broadly used for surgical sutures. In recent decades, silk has also gathered interest for the production of bioscaffolds in tissue engineering. In particular, for VTE, it demonstrates interesting advantages such as controllable biodegradation, low immunogenicity, extraordinary mechanical strength, and wide scaffolding applications, as it can be used in the form of films, hydrogels, nanofibers, and nanoparticles. Moreover, it is an easily accessible material, both eco-sustainable and low cost. Mostly, silk fibroin from mulberry silkworm has been studied for scaffold production [18,134,135].

In 2020, Rodriguez et al. produced silk gel-spun TEVGs with a controlled ultrastructure. By varying the viscosity of the silk solutions, they obtained grafts with different pore sizes and porosity until higher porosities were achieved. This led to scaffolds showing faster degradation times, native-like mechanical properties, and the ability to withstand surgical suturing. Moreover, the highly porous silk material demonstrated satisfactory in vivo biocompatibility in rats, with cell integration; however, patency was not achieved; thus, even though the material proved to be tunable, combination with other materials was suggested [136]. Tanaka et al. produced silk-based small-diameter TEVGs by braiding silk fibroin. They tested the grafts in vivo in mice by performing the cuff technique for implantation; results showed long term patency (up to 6 months), endothelialization, and SMC integration, while macrophages had also invaded the graft. This work permitted comprehension of the silk small-diameter TEVGs’ remodeling process in vivo [137]. In 2023, Durán-Rey et al. evaluated different methods of producing silk fibroin scaffolds. Films by solvent casting, porous membranes by salt leaching, and electrospun scaffolds were produced. While all three silk material forms showed good biocompatibility, favoring EC proliferation, the structural properties of the electrospun membranes allowed better cell guidance and mechanical properties. Thus, their study demonstrated how silk is suitable in different forms for VTE applications, but structural differences lead to different outcomes [138].

Cellularized silk TEVGs have also been evaluated. Rizzi et al. explored bioreactor-based cellularization on electrospun silk scaffolds and, subsequently, dynamic stimulation for culturing. The aim was to create a TEVG with an EC physiological layer in order to achieve biomimicry of the graft, and the results showed that with this method, silk could be optimally endothelialized, paving the way for future studies and possibly personalized scaffold cellularization using autologous EC sources [83].

Finally, silk has also often been combined with other materials. Shi et al. evaluated new silk grafts with improved mechanical properties. First, the silk was methacrylated to improve hydrophilicity, and then it was combined with GelMa in order to enhance its mechanical properties. The obtained hydrogel showed increased breaking strength, elastic modulus, and flexibility. Moreover, the composite hydrogels where shown to be biocompatible and stable, both in vitro and in vivo, using mouse models; in addition, they promoted angiogenesis [139].

In general, silk has been demonstrated to be a suitable biomaterial for VTE. While different processing forms can lead to either mechanical properties that are too weak or scaffolds that are too rigid, these problems can be overcome while biocompatibility and possibility of cellularization are maintained.

### 3.6. Chitosan

Chitosan is a linear polysaccharide derived from chitin’s partial deacetylation, found in the exoskeleton of arthropods. Structurally, chitosan is very similar to glycosaminoglycans contained in the ECM, and its physical properties, such as viscosity or biodegradability, mainly depend on its molecular weight and degree of deacetylation. As a biomaterial, chitosan is used mostly in the form of hydrogel and presents several advantages: it is easily sterilisable, low cost, bioactive, and highly hydrophilic. Its degradability can be controlled, and, notably, it has antibacterial and antifungal properties. Thus, it was also explored as a scaffold for TEVGs production. However, its mechanical properties are far from resembling those of native blood vessels; to overcome this limitation, chitosan is often reticulated with other polymers [24,140,141].

In 2019, Wang et al. evaluated chitosan and carboxymethyl chitosan, respectively, to provide electrospun PCL with antibacterial and antithrombotic properties. They produced bilayered electrospun TEVGs with PCL and chitosan in the outer layer and PCL and carboxymethyl chitosan in the inner layer, and their results demonstrated that the addition of these components promoted endothelialization, while inhibiting thrombosis, and bacterial-killing properties. Moreover, the asymmetric nature of the electrospun scaffolds demonstrated mechanical properties that were superior to native scaffolds [142]. Fiqrianti et al. evaluated the addition of different chitosan concentrations in a blend of collagen and poly-L-lactic acid (PLLA) for electrospinning. The results showed that the addition of the natural components aided hemocompatibility and biocompatibility, while mechanical properties, such as tensile strength and burst pressure, were maintained [143]. Finally, Yin et al. evaluated electrospun poly-L-lactic acid-co-ε-caprolactone (PLCL) with PEGylated chitosan in large in vivo canine models. They demonstrated how the graft allowed cell integration, graft stability up to 6 months after implantation, induction of angiogenic factors, and non-significant calcification after scaffold degradation [144].

Chitosan shows unique antibacterial and antithrombogenic properties; therefore, it is used in VTE to improve these aspects when they are lacking in other materials. However, it remains difficult to find uses for chitosan as a sole material for TEVG production. With further innovations in manufacturing and modifications, technologies may bring research closer to improving its use for VTE.

### 3.7. Decellularized Extracellular Matrix

Previously, decellularized vessels were explored as a means to obtain a tubular scaffold directly from the vessel, and this strategy has led to the development of several vascular grafts, which have indeed reached clinical trials. However, decellularized extracellular matrix (dECM) derived from other tissue sources can also be used for scaffold production. In particular, after decellularization, the matrix can be further processed in order to be solubilized, then used as hydrogels, bioinks, or electrospinning solution. dECM is one of the most bioactive materials that can be found, as it is the one that most closely resembles the native ECM environment; therefore, it has been widely studied for many tissue engineering applications. In VTE, for the same reasons, it has also been widely used, both alone and in combination with other materials, to enhance their mechanical properties and provide them with superior bioactivity [145,146,147].

In 2022, Kamaraj et al. were able to develop a bioink from varicose vein dECM cellularized with ECs derived from the same vein sample and mesenchymal stem cells. The obtained TEVG showed stem cell differentiation towards SMCs and the ability to drive immune cells towards a regenerative phenotype. They were the first to demonstrate that a vascular graft could be derived from the same sample, using both cells and biomaterials, paving the way for future patient-specific, highly bioactive TEVG production [148].

As mentioned, dECM has also been used to functionalize other materials, to enhance their bioactivity. For instance, Gao et al. produced a vascular graft by 3D printing vascular tissue-derived dECM with alginate. The bioink was further loaded with microspheres containing atorvastatin and endothelial progenitor cells. The printing technique also allowed for precise control of the TEVG structure, which demonstrated the ability to induce cell differentiation and showed the ability to treat ischemic limbs in an in vivo mouse model [149]. In another study, a trilayered TEVG was produced using electrospun dECM and PLCL loaded with salidroside. The obtained graft showed interesting mechanical properties and endothelialization potential, both in vitro and in vivo, using rats, with antithrombotic properties and ECM deposition [150]. Gao et al. functionalized dECM scaffolds by coating the surface with PEG, the heparin-chitosan, to enhance mechanical properties, antithrombogenicity, and bacteria-killing abilities. The resulting vascular construct demonstrated these properties, promoted endothelialization, and maintained patency up to 5 months after in vivo implantation in porcine models [151].

Although dECM is commonly used in the form of decellularized vessel scaffolds, it can also be processed for electrospinning and 3D printing. Even though this is an interesting technique, the multiple necessary steps needed to obtain the product (decellularization, solubilization, postprocessing, and manufacturing) make the use of dECM in forms other than native vessels more complicated for VTE.

Thus, natural materials have been recently employed for VTE, with successful results, leading to many studies ending with in vivo studies. Table 2 summarizes the recent research studies evaluated in this work.

## 4. Conclusions

In a context where CDVs are the main cause of death in the world and vessel substitution or bypass are often required, VTE has been shown to be a promising alternative to autologous vascular grafts. Although it is a challenge to replicate the necessary biological and mechanical properties, great progress has been made in the production of TEVGs. New fabrication techniques, insights into biomaterial design, and innovative tissue maturation strategies have led to improved results. Natural materials have received more attention due to their innate bioactivity, and, thanks to the progress made in the past decades, some of the problems tied to their use have been overcome. In particular, the advances in fabrication techniques have allowed better manipulation and tailorability of natural materials, which were significant challenges in the use of biological materials; at the same time, many research works reported herein also demonstrated results in obtaining natural-based TEVGs with appropriate mechanical properties for blood vessel replacement. Astonishingly, many research works even reported the production of small-caliber TEVGs made with natural materials, an achievement that has challenged researchers for many years. These innovations have also led to many works obtaining promising results with in vivo experimentation using natural-based TEVGs. Biomaterials like collagen and elastin remain the top choices when it concerns biomaterials for VTE; some collagen products have reached the market, such as Artegraft^®^ or ProCol^®^, while new manufacturing techniques, such as electrospinning and 3D printing, are taking over and being used more frequently to obtain highly controlled graft ultrastructures. However, challenges with the use of all-natural TEVGs still remain, such as the ability to obtain functional and effective vascular grafts (encompassing both biological and mechanical properties, made of a single and natural component) and if—and when—most of these will be able to achieve commercialization. However, considering the positive advances reported herein, TEVGs from natural material scaffolds show potential for being translated from research to clinical practice in the near future, while other natural TEVGs, such as cell-derived TEVGs or hybrid TEVGs, have already been utilized in human trials [152,153,154].

## Figures and Tables

**Table 1:** Examples of recent biomaterials, cells, fabrication techniques, and tissue maturation methods for functional TEVG production.

Biomaterial	Cell Type	Fabrication Technique	Tissue Maturation	Highlights	Year	Refs.
PCL	Human endothelial colony forming cells and multipotent mesenchymal stromal cells	Electrospinning and melt electrowriting	Perfusion bioreactor combining static maturation on outside layer and luminal shear stress dynamic stimulation	The bilayered TEVG showed a physiological-like cell organization and phenotype, due to the bioreactors design which allows the achievement of vascular layer-specific characteristics.	2019	[84]
Gelatin coated PGA	vSMCs derived from hiPSCs	Cell seeding on premade biodegradable scaffolds	Peristaltic pump bioreactor for incremental pulsatile stretching dynamic culturing	The hiPSCs-derived vSMCs seeded on the biodegradable scaffold produced cellularized collagenous TEVGs with physiological-like mechanical properties, which were maintained, along with patency, following in vivo implantation.	2020	[85]
ECM and PCL	Acellular	Decellularization and electrospinning	None	Small-diameter TEVG made by electrospinning PCL for reinforcing a decellularized vessel. The graft showed good integration between the materials, biocompatibility, and hemocompatibility.	2020	[86]
Polydioxanone and PCL	Acellular	Electrospinning and 3D printing	None	This bilayered TEVG, enriched with immobilized VEGF, proved to be a good conduit for vascular tissue regeneration, allowing for improved cellularization in vivo and in vitro. Moreover, it was able to maintain mechanical properties after in vivo implantation, due to the 3D-printed PCL reinforcement.	2020	[87]
Polyurethane	Acellular	Dip-coating on 3D-printed vascular templates	None	The synthetic graft showed excellent physiological-like mechanical properties, surpassing those of commercially available grafts. Furthermore, the TEVG proved to reduce thrombogenesis in vivo, with improved endothelialization of the graft.	2021	[88]
ECM	Acellular	Decellularization	None	A new decellularization method was developed to ensure antigen removal in the TEVG, with retention of ECM basement membrane. This allowed the achievement of a TEVG for small-diameter grafts with high patency rates after in vivo implantation.	2021	[89]
Alginate and collagen	Acellular	Molding	None	Natural-based TEVGs with tunable macro-architecture properties were produced. The cross-linking method developed proved to improve stability and mechanical properties while maintaining bioactivity.	2022	[90]
PCL and ECM	Acellular	Electrospinning	None	The TEVG, with heparin and VEGF added, showed excellent hemocompatibility and cell infiltration. Moreover, in vivo studies demonstrated the TEVGs‘ integration with a decreased thrombus risk.	2022	[91]
PCL	Murine vSMCs	Electrospinning	Perfusion-based bioreactor for seeding and culturing cells under dynamic conditions	The use of a low-cost and simple dynamic cell seeding and culturing bioreactor proved to produce a TEVG with more evenly distributed and viable cells compared to static conditions.	2022	[92]
PCL, collagen, and gelatin	Acellular	Electrospinning	None	An electrospun trilayered TEVG made with an inner PCL/collagen layer to improve endothelialization, a medial PCL layer, and an outer PCL/gelatin layer. The construct showed physiological-like ultrastructure of electrospun fibers and mechanical properties exceeding those of native vessels.	2022	[93]
Polyurethane, silk fibroin, gelatin, and chitosan	Acellular	Electrospinning and freeze-drying	None	Heparinized multicomponent TEVGs showed increased mechanical properties, cell integration, and ability to release heparin over time, producing antithrombotic characteristics.	2022	[94]
Alginate and collagen	Murine fibroblasts	3D printing	None	The addition of collagen to the bioink proved to ameliorate the mechanical properties of the construct and increase cell adhesion and viability.	2022	[95]
Silk fibroin and polyurethane	Acellular	Electrospinning	None	Hybrid TEVGs, with physiological-like structure characteristics, were obtained. The small-calibre TEVGs showed good compliance, with adequate application up to 3 months after in vivo implantation.	2022	[96]
Alginate, hyaluronic acid, and ECM	Acellular	3D printing	None	The approach produced a multi-component bioink that could be printed into a vascular graft with appropriate mechanical properties. Moreover, the TEVG also showed excellent angiogenic and anti-inflammatory activity in vitro.	2023	[97]

**Table 2:** Summary of recent research work involving natural materials for VTE.

Biomaterial	Study	Outline	Year	Refs.
Collagen	In vitro	A trilayered cellularized physiological-like TEVG produced by molding and dynamic maturation, showing native vessel-like mechanical properties.	2022	[107]
	In vitro	Bilayered and cellularized TEVGs made using coaxial extrusion, with high collagen concentrations for increased mechanical properties.	2022	[108]
	In vitro	A highly tailorable densified collagen construct with enhanced stability and mechanical properties and possibility of cellularization.	2023	[109]
	In vitro	Electrospun PCL/collagen/heparin TEVGs with ameliorated flexibility and bursting strength compared to native vessels.	2022	[110]
	In vitro/in vivo	Enzyme-laden hyaluronic acid/collagen/PCL electrospun scaffold favoring endothelialization and antithrombogenicity.	2022	[111]
Gelatin	In vitro	3D-printed GelMa constructs stabilized by dual cross-linking showing enhanced mechanical properties and endothelialization.	2021	[113]
	None	A novel additive lathe printing method to achieve highly tunable GelMA tubular structures for VTE.	2023	[114]
	In vitro/in vivo	Electrospun gelatin cross-linked with oxidized carboxymethyl cellulose showing excellent biocompatibility both in vitro and in vivo.	2017	[115]
	In vitro	Gelatin was electrospun with PCL and PGE to increase mechanical properties and tailor ultrastructure, achieving cell adhesion and migration in the scaffold and edothelialization.	2017	[116]
	In vitro	Electrospun PCL, PGLA, and gelatin with controlled fiber orientation showing increased guidance for cell orientation and appropriate mechanical properties.	2020	[117]
Fibrin	In vitro/in vivo	Electrospun PU/fibrin small-caliber TEVGs showed optima biocompatibility and mechanical properties, with graft patency and thrombosis risk reduction achieved up to 3 months after implantation.	2020	[120]
	In vitro/in vivo	Electrospun PCL/fibrin grafts with increased mechanical properties demonstrated good hemocompatibility and biocompatibility.	2020	[121]
	In vivo	Electrospun PCL/fibrin small-caliber grafts studied in vivo up to 9 months, showed ability to induce neoartery regeneration.	2021	[122]
	In vitro/in vivo	Fibrin graft embedded with heparin for decreased thrombogenicity and showed stability after up to 12 months of storage.	2022	[123]
	In vitro/in vivo	Fibrin-based decellularized TEVG from ovine fibroblasts showed graft recellularization and good patency up to 6 months after implantation in ovine model.	2014	[124]
	In vitro/in vivo	Fibrin-based decellularized TEVG from human fibroblasts demonstrated no immune reactions, graft recellularization, and stability up to 6 months after implantation in baboon model.	2017	[125]
Elastin	In vitro	Self-assembling functionalized elastin scaffold able to limit platelet adhesion and activation, promote endhotelialization, and induce SMCs’ contractile phenotype.	2023	[129]
	In vitro	A multilayered elastin/collagen graft with highly controlled ultrastructure, showing good SMC biocompatibility and low immunogenicity.	2020	[130]
	In vitro	Molded cellularized collagen grafts with functionalized ELR addition demonstrated improved elastic-mechanical properties and cell functionality.	2020	[131]
	In vitro/in vivo	The addition of elastin to the silk fibroin scaffolds improved mechanical properties and cell adhesion, maintaining patency and bioactivity after implantation.	2021	[132]
	In vitro/in vivo	Tropoelastin lamellae embedded in PSG electrospun scaffolds led to formation of neoartery 8 months after in vivo implantation.	2022	[133]
Silk	In vivo	Tunable gel spun silk TEVGs with high porosities showed improved mechanical properties and good cellularization after in vivo implantation.	2020	[136]
	In vivo	Small-diameter braided silk fibroin grafts were used to understand graft remodeling after implantation, showing excellent biocompatibility and long-term spotipatency.	2020	[137]
	In vitro	Physico-chemical characterization of 3 different silk biomaterials was performed, all showing good biocompatibility for VTE applications.	2023	[138]
	In vitro	Cellularized silk electrospun grafts with dynamic stimulation for physiological-like EC monolayer.	2022	[83]
	In vitro/in vivo	Methacrylated silk and GelMa hydrogels showing enhanced mechanical properties, biocompatibility, and angiogenic potential both in vitro and in vivo.	2023	[139]
	In vitro	Bilayered electrospun chitosan and PCL grafts with antithrombogenic and antibacterial properties; in addition, demonstrated rapid endothelialization.	2019	[142]
Chitosan	In vitro	Chitosan-rich collagen/PLLA TEVGs showed improved hemocompatibility and biocompatibility.	2018	[143]
	In vitro/in vivo	Evaluation of chitosan/PLCL vascular grafts in large animal model demonstrated stability and biocompatibility up to 24 weeks.	2020	[144]
Decellularized extracellular matrix	In vitro	A bioink made of dECM and ECs derived from the same vein sample, supplemented with mesenchymal stem cells showing ability to induce cell differentiation.	2022	[148]
	In vitro/in vivo	dECM and alginate bioink, cellularized with endothelial progenitor cells, showing bioactivity and therapeutic potential for ischemic disease.	2017	[149]
	In vitro/in vivo	Electrospun dECM and PLCL loaded with salidroside demonstrated bioactivity with good endothelialization and ECM deposition in vitro and in vivo.	2023	[150]
	In vitro/in vivo	dECM scaffold modified with PEG, heparin, and chitosan showed appropriate mechanical properties and long-term patency in large in vivo model.	2022	[151]

## Data Availability

Not applicable.
